# Prognostic factors of poor outcomes in pneumonia in older adults: aspiration or frailty?

**DOI:** 10.1007/s41999-023-00929-0

**Published:** 2024-02-03

**Authors:** Yuki Yoshimatsu, Heledd Thomas, Trevor Thompson, David G. Smithard

**Affiliations:** 1 Elderly Care, Queen Elizabeth Hospital, Lewisham and Greenwich NHS Trust, Stadium Rd, London, SE18 4QH UK; 2 Centre for Exercise Activity and Rehabilitation, School of Human Sciences, University of Greenwich, London, UK; 3 Scientific Research WorkS Peer Support Group (SRWS-PSG), Osaka, Japan; 4 Centre for Chronic Illness and Ageing, University of Greenwich, London, UK

**Keywords:** Dysphagia, Aspiration pneumonia, CAP, Frailty, Anticholinergic

## Abstract

**Purpose:**

Little is known about the long-term and functional prognoses of older adults with pneumonia, which complicates their management. There is a common belief that aspiration is a poor prognostic factor; however, the diagnosis of aspiration pneumonia (AP) lacks consensus criteria and is mainly based on clinical characteristics typical of the frailty syndrome. Therefore, the poor prognosis of AP may also be a result of frailty rather than aspiration. This study investigated the impact of AP and other prognostic factors in older patients with pneumonia.

**Methods:**

We performed a retrospective cohort study of patients aged 75 years and older, admitted with pneumonia in 2021. We divided patients according to their initial diagnosis (AP or non-AP), compared outcomes using Kaplan–Meier curves, and used logistic regression to identify independent prognostic factors.

**Results:**

803 patients were included, with a median age of 84 years and 52.7% were male. 17.3% were initially diagnosed with AP. Mortality was significantly higher in those diagnosed with AP than non-AP during admission (27.6% vs 19.0%, *p* = 0.024) and at 1 year (64.2% vs 53.1%, *p* = 0.018), with survival analysis showing a median survival time of 62 days and 274 days in AP and non-AP, respectively (*χ*^2^ = 9.2, *p* = 0.002). However, the initial diagnosis of AP was not an independent risk factor for poor prognosis in multivariable analysis. Old age, frailty and cardio-respiratory comorbidities were the main factors associated with death.

**Conclusion:**

The greater mortality in AP may be a result of increased frailty rather than the diagnosis of aspiration itself. This supports our proposal for a paradigm shift from making predictions based on the potentially futile labelling of AP or non-AP, to considering frailty and overall condition of the patient.

**Supplementary Information:**

The online version contains supplementary material available at 10.1007/s41999-023-00929-0.

## Key summary points

**Aim:**

The aim of this study was to investigate the impact of aspiration pneumonia and other prognostic factors that affect long-term and functional outcomes in older patients with pneumonia.

**Findings:**

Mortality was significantly higher in aspiration pneumonia than non-aspiration pneumonia during admission and at 1 year. However, independent risk factors for poor prognosis were old age, frailty and cardio-respiratory comorbidities and not the initial diagnosis of aspiration pneumonia.

**Message:**

The management of older adults with pneumonia must be based on frailty and overall condition rather than the potentially futile labelling of aspiration pneumonia or non-aspiration pneumonia.

**Supplementary Information:**

The online version contains supplementary material available at 10.1007/s41999-023-00929-0.

## Introduction

As Sir William Osler, the founding father of modern medicine, wrote in his first medical textbook in 1892, pneumonia is often regarded as ‘the special enemy of old age’ [1]. By the third edition of his book, he rephrased this to the well-known quote: “Pneumonia may well be called the friend of the aged” [2]. Interestingly, these contradictory statements both describe the same characteristic of pneumonia in older adults: its high mortality. Despite revolutionary advances in the diagnosis, treatment and prevention of pneumonia, the comparatively high mortality of pneumonia in older adults has remained unchanged.

Predicting the prognosis of older patients with pneumonia is essential for the optimal treatment and, where appropriate, a palliative approach [3]. However, the multifactorial pathophysiology of pneumonia in older adults makes prediction difficult. Traditional pneumonia severity indicators recommended by guidelines such as CURB-65 [4], pneumonia severity index (PSI) [5], and A-DROP [6] have been reported to be less useful in this population [7–9]. In pneumonia in older adults, where up to 90% of cases may be aspiration-related [10], the severity of the lung inflammation itself is not the sole determinant of prognosis. Rather, their prognosis is a result of a complex combination of comorbidities [11, 12], frailty, sarcopenia, malnutrition, poor oral hygiene, oral frailty, and impaired immune responses. This is why we previously proposed the term ‘Frailty associated pneumonia (FAP)’ [13]. Some reported poor prognostic factors include multimorbidity, aspiration, reduced cough effectiveness, malnutrition, older age, higher dependency, loss of muscle mass and anticholinergic drugs [12–24]. No clear prognostic factors have been identified for pneumonia in older adults, despite the high disease prevalence and mortality.

In our previous studies, we revealed a lack of unified diagnostic criteria for aspiration pneumonia (AP) in both literature [25] and clinical practice [26], and the reality that older adults with pneumonia were labelled as AP or non-AP depending on comorbidities and baseline frailty [26]. Their management differed according to the initial diagnosis; patients diagnosed with AP were less likely to be investigated, but more likely to be made nil by mouth (NBM) and treated with broad-spectrum antibiotics, whilst those diagnosed with non-AP were more likely to be investigated for alternative diagnoses. These initial diagnoses and differences in management may influence outcomes. Although it is generally accepted that the prognosis is worse in AP than non-AP, this may be due to confounding factors such as frailty and comorbidities rather than aspiration itself. Frailty is a particularly important factor affecting prognosis in cancer and heart failure amongst other conditions [27–29]. There is a need to assess AP and its independent impact on prognosis amongst other risk factors including frailty.

Furthermore, when discussing prognosis, it is not only the short-term prognosis that is of interest in clinical practice. Long-term mortality after an episode of pneumonia is also high in older adults [30, 31], and survivors have been reported to be at risk of functional decline [32]. To discuss the optimal care for the patient, it is essential to make an evidence-based prediction of their long-term prognosis and the impact of pneumonia on their quality of lives. Despite the large number of studies on the immediate outcomes of patients with pneumonia, long-term prognosis and functional outcomes are rarely discussed, and there is a lack of large studies based in the UK or European countries.

Therefore, following our initial study on the diagnosis and management of older patients admitted with pneumonia [26], we now report on their prognosis and the impact of AP amongst other associated factors to identify poor prognostic factors, with a particular focus on long-term and functional outcomes.

## Methods

### Study design

This was the one-year follow-up of a retrospective cohort study of patients admitted with pneumonia to Queen Elizabeth Hospital (Lewisham and Greenwich NHS Trust). Ethical approval was obtained from the Lewisham and Greenwich NHS Trust (Number 7211), and informed consent was waived due to the retrospective nature of the study. Detailed inclusion and exclusion criteria, data collection methods, and patient selection process are described in our previous paper [26]. Briefly, we included patients aged 75 years and older admitted with community-acquired pneumonia (CAP) in 2021, excluding those admitted for COVID-19. We collected data on the patient background, comorbidities, presenting condition, and outcome from medical records.

### Data collection

We used the following data collected for our previous study: patient demographics (age, sex), social history (whether they lived at home or in a care/nursing home, and whether they had professional carers or not), medical history (comorbidities, drugs, pneumonia within the past year), presenting condition (CURB-65 score [4], pneumonia severity index (PSI) [5], clinical frailty score (CFS) [33], and initial diagnosis (AP or non-AP). The initial diagnosis was extracted according to what was documented on the consultant physician ward round at the time of admission. This was because our study intended to investigate the reality of how AP was being diagnosed in clinical practice. Non-AP was defined as any patient diagnosed with CAP that was not documented as AP. In addition, we extracted the following outcome data from the medical records: survival at the time of discharge and 1 year after admission, level of dependence at discharge, recurrence of pneumonia within 1 month after admission, and length of hospital stay (days). In the UK, community medical records can be partially accessed from the hospital medical record system, allowing us to assess the patients’ date of death. We categorised the level of dependence into the following five levels, with 1 being the least dependent and 5 being the most dependent: (1) living at home alone, (2) living at home alone with arranged professional carers, (3) living with family (with/without arranged professional carers), (4) living in a residential home, and (5) living in a nursing home. The need for increased support (moving from a lower to a higher category) was defined as ‘increased level of dependence’. Amongst the potential risk factors for poor outcomes, we selected the following, based on the results of our previous study [26] and other reports: age, sex, social history (according to the level of dependence explained above), neurological condition (stroke or degenerative neurological disorder/disease), dementia, cardiac condition (ischaemic or congestive heart disease), diabetes mellitus, chronic respiratory disorder, CRIDECO anticholinergic load scale (CALS), history of pneumonia within 1 year prior to admission, CFS, CURB-65, initial diagnosis of AP, being put on nil by mouth (NBM) on admission, and a speech and language therapist (SLT) referral.

### Statistical analyses

We used chi-square tests to compare AP and non-AP diagnostic groups for the outcomes of mortality (overall, in-hospital and after discharge up to one year from admission), 30-day pneumonia recurrence and increased dependency level, and a t-test for the continuous outcomes of length of stay (days). We performed survival analysis for mortality at 12 months, and compared median survival time across AP and non-AP diagnoses with Kaplan–Meier curves.

Logistic regression was used to calculate adjusted estimates for the AP diagnostic group and to identify additional prognostic factors that were included in the covariates listed in the Data Collection section. We also conducted multiple linear regression on the continuous outcome of length of hospital stay (days). Analyses were performed using Microsoft Excel and the *survival* and *survminer* packages [34, 35] in R. A *p* value < 0.05 was considered to be statistically significant for all analyses.

## Results

### Patient background

A total of 803 patients were included in the study. Demographical data are shown in Table 1. There were 423 males (52.7%), with a median age of 84 years (interquartile range: 80–89). One hundred and thirty-nine patients (17.3%) were initially diagnosed with AP. As discussed in our previous study, AP tended to be diagnosed on the basis of frailty and comorbidities rather than the assessment of swallowing, cough or oral hygiene [26]. This was derived from the fact that the initial diagnosis of AP or non-AP was done prior to any assessments by SLTs, and there was no mention of swallow screening tests or oral hygiene status before or at the timing of pneumonia diagnosis. Other details on patient background and intervention are explained in our previous study [26].

**Table 1:** Patient background and management

Factor	AP (*n* = 134)		non-AP (*n* = 669)		*p* value
	*n*	%, IQR	*n*	%, IQR	
**Background**					
Male (*n*, %)	72	53.7	351	52.5	0.766
Age (median, IQR)	85	80–90	84	80–89	0.113
Clinical frailty scale (median, IQR)	6	5–7	5	4–6	< 0.001
**Dependence level before admission**					< 0.001
Living at home alone (*n*, %)	5	3.7	109	16.3	
Living at home alone with professional carers (*n*, %)	28	20.9	140	20.9	
Living with family (*n*, %)	61	45.5	344	51.4	
Living in a residential home (*n*, %)	26	19.4	48	7.2	
Living in a nursing home (*n*, %)	14	10.4	28	4.2	
**Past medical history, comorbidities**					
Neurological condition (*n*, %)	49	36.6	122	18.2	< 0.001
Dementia (*n*, %)	69	51.5	154	23.0	< 0.001
Ischaemic/congestive cardiac condition (*n*, %)	31	23.1	207	30.9	0.071
Type 2 diabetes mellitus (*n*, %)	22	16.4	161	24.1	0.054
Respiratory disorder (*n*, %)	22	16.4	198	29.6	0.002
Pneumonia within 1 year (*n*, %)	38	28.4	135	20.2	0.036
CALS (median, IQR)	1	0–2	1	0–3	0.491
**Presenting condition and actions taken**					
CURB-65 (median, IQR)	2	2–3	2	1–2	< 0.001
Nil by mouth orders (*n*, %)	70	52.2	49	7.3	< 0.001
SLT referral (*n*, %)	94	70.1	119	17.8	< 0.001

*AP* aspiration pneumonia, *IQR* interquartile range, *CALS* CRIDECO anticholinergic load scale, *SLT* speech and language therapist

### Outcomes

Differences in outcomes between diagnostic groups are shown in Table 2. The mortality rate during admission was significantly higher in the AP group than in the non-AP group (27.6% vs 19.0%, *p* = 0.024). Similarly, the mortality rate at 1 year after admission was significantly higher in the AP group than in the non-AP group (64.2% vs 53.1%, *p* = 0.018). There was no significant difference between the groups for death after discharge, increased dependency, 30-day pneumonia recurrence, or length of hospital stay.

**Table 2:** Patient outcomes according to the initial diagnosis

Outcome	AP (*n* = 134)	non-AP (*n* = 669)	*p* value
Death in hospital (*n*, %)	37/134 (27.6)	127/669 (19.0)	0.024
Death at 1 year, after discharge (*n*, %)	49/97 (50.5)	228/542 (42.1)	0.122
Death (total) at 1 year (*n*, %)	86/134 (64.2)	355/669 (53.1)	0.018
Pneumonia recurrence within 30 days (*n*, %)	15/97 (15.5)	69/543 (12.7)	0.459
Increased dependence at discharge (*n*, %)	25/97 (25.8)	124/542 (23.4)	0.555
Length of hospital stay (days) (median, IQR)	8 (5–15)	8 (4–14)	0.518

*AP* aspiration pneumonia, *IQR* interquartile range

### Risk factors for poor outcome

The full results of the logistic regression are shown in the supplementary table. In summary, the odds of in-hospital death were significantly higher for older age, history of pneumonia in the previous year, presence of cardiac condition, higher CURB-65 score and NBM on admission. Amongst survivors at discharge, the odds of death within 1 year of admission were significantly higher for older age, respiratory condition, and a higher CFS score. The odds of death within 1 year of admission overall were significantly higher for older age, respiratory condition, a history of pneumonia in the previous year, a higher CFS score, a higher CURB-65 score, being made NBM on admission, and having a speech therapy referral. A diagnosis of AP (as opposed to non-AP) was not an independent risk factor of either poor outcome after controlling for severity, comorbidities and age.

We performed analysis regarding the dependency level on 640 patients who had not died in the hospital and found that the odds of increased dependency at discharge were significantly higher depending on a higher CFS score, higher CURB-65 score, being made NBM on admission, and having an SLT referral. On the other hand, higher dependence at admission and neurological conditions may be related with a more favourable outcome. Again, a diagnosis of AP was not an independent risk factor for poor functional outcome.

Likewise, we performed an analysis of pneumonia recurrence in the 639 patients who were discharged alive within 30 days. The odds ratio for pneumonia recurrence within 30 days of admission was significantly higher in those with a pneumonia history in the previous year, and those with an SLT referral. A diagnosis of AP was not amongst the independent risk factors.

**Fig. 1:**
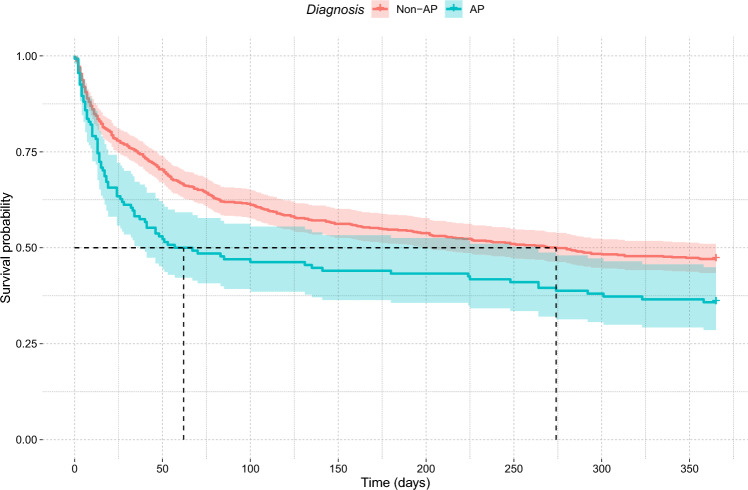
Survival curve according to initial diagnosis. The median time to death was 62 days in the AP group and 274 days in the non-AP group, with most deaths occurring in the first few weeks after admission

Kaplan–Meier curves for 1-year survival are shown in Fig. 1, which revealed a median of 274 days to death for a non-AP diagnosis and 62 days for an AP diagnosis (*χ*^2^ = 9.2, *p* = 0.002), with survival curves indicating that this appeared to be primarily a result of a more rapid death rate associated with an AP diagnosis in the early stages after admission.

**Fig. 2:**
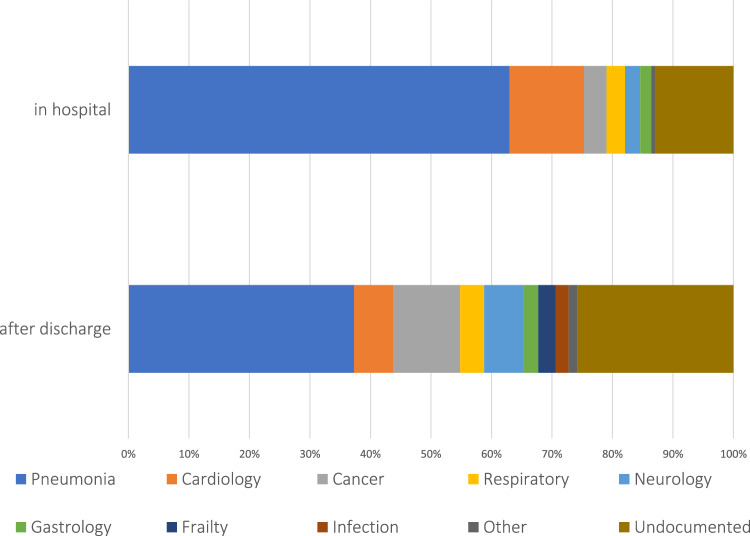
Causes of death according to the timing of death. The most common causes of death in hospital were pneumonia and cardiologic conditions, whereas for death after initial discharge, although pneumonia was still the most common cause of death recorded, there were more cancers and frailty than cardiologic causes

Figure 2 shows the causes of death in the hospital and after discharge (within 1 year). The leading cause of death in both groups remained pneumonia, whilst the second most common cause of death in hospital was cardiologic conditions such as ischaemic heart disease and congestive heart failure. Causes that increased after discharge included more chronic conditions such as cancer, frailty and neurological disorders. There were no significant differences in the causes of death between the AP and non-AP groups.

## Discussion

In older adults with pneumonia, whilst age was understandably a common risk factor for poor prognosis, independent risk factors differed between outcomes. Frailty was a common independent risk factor for poor long-term and functional outcomes. These data provide valuable insight into the prediction of patient-relevant outcomes and enable more informed decision-making regarding their care.

The most important finding to highlight is that although patients diagnosed with AP had significantly worse survival than those with non-AP (Fig. 1), this diagnosis was not an independent risk factor in any of the five outcomes analysed, after controlling for other factors (supplementary table). Rather, age, cardiorespiratory comorbidities, and a higher CFS score were independent risk factors. This suggests that what affects survival is the general condition rather than aspiration. This is consistent with the reality that the diagnosis of AP in older adults has evolved into a diagnosis of frailty, as discussed in our previous studies [13, 25, 26], and other related studies [36]. Clinicians tend to provide a clinical diagnosis of AP in the more severely frail and those with more comorbidities; hence it is natural that patients diagnosed with AP have a poor prognosis. Our results highlight the importance of considering the patient’s frailty and comorbidities and not just the diagnostic label when predicting prognosis and deciding management.

The overall mortality rate during admission in older adults hospitalised with CAP was 20.4%, which is higher than the younger population [37]. Older age and cardiac comorbidities are previously reported risk factors for poor prognosis, whilst a history of pneumonia in the previous year is a new important risk factor. Those who were NBM on admission had a high OR of 4.96, likely reflecting their severe condition on admission and not necessarily a prognostic factor. Similarly, the effects of CURB-65 must be interpreted with caution, as there was no difference in the median CURB-65 score amongst the two groups. Interestingly, dementia was associated with a favourable short-term prognosis. This may be partly because admission in those with a history of dementia is driven by delirium rather than the severity of pneumonia.

During the first year after admission, 54.9% of patients died. Even after being discharged alive, 43.3% had died in the first year after admission. This is a significant figure that must be borne in mind when managing these patients. The survival curve in Fig. 1 shows that most of these deaths occurred within the first few weeks of admission. Those diagnosed with AP had a significantly worse prognosis, but interestingly, after a few months, the survival curves of AP and non-AP appear to become parallel. A similar dissociation of survival curves between AP and non-AP was also observed in a recent study in France [38], in which frailty was not compared between groups. It can be spectated that frail patients (who were more likely to be diagnosed with AP) were more likely to die after an acute episode of pneumonia, as shown in Fig. 2 where frailty and neurological conditions were amongst the common causes of death after discharge.

Factors that adversely affected long-term survival were older age, respiratory disorder, and a higher CFS score, of which the latter two were not associated with short-term survival, indicating that frailty and comorbidities affect the survival of patients after recovery from pneumonia, regardless of the severity of pneumonia.

Pneumonia recurred within 30 days of admission in 13% of those who had initially recovered. A history of pneumonia in the previous year and SLT referral were independently associated with recurrence, but other factors commonly considered to be risk factors (such as AP or diabetes mellitus) were not.

Amongst survivors, 23.3% had an increased dependence level at discharge. Independent risk factors related to increased dependence level were frailty, more severe pneumonia, and SLT referral. On the other hand, a higher dependence level at admission and neurological conditions were related to favourable outcomes. This may be because these patients already had a high dependence level (living with family or carers). It is clinically important to identify patients at risk of functional decline early and initiate physiotherapy to avoid preventable loss of mobility [39, 40].

These study findings have many clinical implications. The poor prognosis of older patients admitted with pneumonia must be kept in mind, with one in five patients dying during the hospital stay, and a quarter of survivors being discharged with increased dependence, and nearly half of survivors dying within the next year. Whilst short-term survival is the imminent factor for clinicians when treating a patient with pneumonia, it is not the only concern for patients and their families. When predicting prognosis and communicating future outlook and management options with patients and their families, clinicians must consider increased dependence and long-term prognosis too. Discussion of treatment escalation plans and community care would be particularly important for more frail patients and those with comorbidities such as respiratory conditions, and a multidisciplinary team approach is the key to adequate management and shared-decision making.

Another important finding is that the general condition of the patient seems to independently affect the outcome more than the diagnosis of AP itself. This supports our previous proposal of a paradigm shift in the management of pneumonia in older adults, from the potentially futile labelling of AP or non-AP to considering frailty and the overall condition of the patient [26]. However, this is a preliminary interpretation from an observational study and would need to be confirmed by a randomised controlled trial before any further conclusions could be drawn. In the meantime, it is safe to say that our common practice of making patients NBM is best considered carefully, depending on the individual patient’s condition. Eating and drinking are a vital source of nutrition and hydration for these patients, as well as being an important factor in their quality of lives. Patients should be assessed for swallowing and oral hygiene and encouraged to continue oral intake as appropriate.

These findings also have research implications, as the long-term prognosis and prognostic factors are not yet known. With advances in electronical medical records and technology, larger database studies looking at long-term prognosis are needed.

There were limitations associated with this study, mainly owing to the single-centre, retrospective design. In particular, data on deaths after discharge may not have been available in the medical record system if they occurred outside of the local NHS Trust, meaning that there may have been more deaths. However, this was a relatively large study with an initial list of over 1400 patients with pneumonia, from a large acute hospital. The majority of older adults admitted will continue to be treated by the same NHS Trust after discharge. There have been no similar studies of older adults diagnosed with pneumonia in the UK, highlighting the importance of this study. We believe this is a meaningful step in addressing the challenging conundrum of the diagnosis and management of AP. In future studies, it would be useful to assess data prospectively and assess frailty in a more robust and accurate way.

## Conclusion

AP was associated with much greater mortality, with survival analysis showing a median survival of 62 days compared to 274 days in the non-AP group. However, multivariable regression showed that greater mortality in AP may be a simple function of increased frailty rather than the diagnosis of aspiration itself. Older adults with a diagnosis of pneumonia not only have a poor short-term prognosis but also poor long-term and functional outcomes. Survivors are at high risk of becoming increasingly dependent, and many die within the following months. This supports our proposal for a paradigm shift in the focus of clinicians managing older adults with pneumonia, from making predictions and decisions based on the potentially futile labelling of AP or non-AP, to considering frailty and overall condition of the patient. It is crucial to consider patient-relevant outcomes and associated risk factors when managing older adults with pneumonia.

### Supplementary Information

Below is the link to the electronic supplementary material.

Supplementary file1 (DOCX 18 KB)

## Data Availability

All data are applicable in the paper.
