# Supplementary material for: Prognostic factors of poor outcomes in pneumonia in older adults: aspiration or frailty?
Source: Eur Geriatr Med. 2024 Feb 3;15(2):481–8. doi: 10.1007/s41999-023-00929-0 (PMC10997696; doi:10.1007/s41999-023-00929-0)
Supplement: Supplementary file 1 — Supplementary file1 (DOCX 18 KB) [file 41999_2023_929_MOESM1_ESM.docx]

**Supplementary table. Factors related with outcomes**

|  | **Death in hospital** | | **Death after**  **discharge, < 1y** | | **Death (total),**  **< 1y** | | **Increased dependence** | | **30-day recurrence** | | **length of stay** | |
| --- | --- | --- | --- | --- | --- | --- | --- | --- | --- | --- | --- | --- |
|  | OR [95%CI] | p | OR [95%CI] | p | OR [95%CI] | p | OR [95%CI] | p | OR [95%CI] | p | B [95%CI] | p |
| **age (per year)** | 1.05 [1.02,1.08] | .003 | 1.03 [1.00,1.06] | .023 | 1.05 [1.02,1.07] | <.001 | 1.02 [0.98,1.05] | .290 | 1.01 [0.97,1.06] | .501 | -0.09 [-0.21,0.04] | .181 |
| **male sex** | 1.04 [0.71,1.54] | .823 | 0.74 [0.52,1.04] | .080 | 0.79 [0.58,1.08] | .140 | 1.12 [0.75,1.66] | .588 | 1.08 [0.66,1.77] | .762 | 0.61 [-0.91,2.13] | .431 |
| **social history** | 1.09 [0.87,1.37] | .457 | 0.96 [0.79,1.17] | .706 | 1.02 [0.85,1.22] | .853 | 0.72 [0.57,0.90] | .005 | 1.01 [0.76,1.35] | .928 | -0.78 [-1.66,0.11] | .085 |
| **neurologic condition** | 1.09 [0.68,1.71] | .725 | 0.68 [0.44,1.05] | .087 | 0.71 [0.48,1.05] | .088 | 0.52 [0.29,0.90] | .024 | 1.47 [0.83,2.57] | .179 | -1.59 [-3.51,0.33] | .105 |
| **dementia** | 0.44 [0.27,0.70] | <.001 | 1.42 [0.93,2.18] | .103 | 1.02 [0.69,1.51] | .915 | 0.65 [0.39,1.08] | .102 | 0.98 [0.54,1.76] | .948 | -1.11 [-3.02,0.80] | .253 |
| **cardiac condition** | 1.56 [1.04,2.34] | .032 | 1.07 [0.74,1.55] | .716 | 1.21 [0.87,1.69] | .269 | 0.68 [0.42,1.07] | .104 | 1.15 [0.67,1.94] | .605 | -0.16 [-1.82,1.49] | .845 |
| **diabetes mellitus** | 0.73 [0.46,1.16] | .194 | 0.99 [0.67,1.46] | .958 | 0.88 [0.62,1.26] | .487 | 0.72 [0.44,1.14] | .172 | 0.86 [0.47,1.49] | .600 | -1.36 [-3.12,0.41] | .131 |
| **respiratory disorder** | 1.07 [0.69,1.65] | .752 | 1.59 [1.08,2.34] | .019 | 1.45 [1.02,2.07] | .040 | 0.84 [0.53,1.32] | .459 | 1.35 [0.78,2.30] | .274 | -1.55 [-3.28,0.19] | .080 |
| **CALS** | 1.03 [0.92,1.14] | .604 | 1.04 [0.94,1.15] | .479 | 1.06 [0.96,1.16] | .255 | 0.96 [0.85,1.07] | .466 | 0.98 [0.85,1.12] | .817 | 0.07 [-0.38,0.52] | .765 |
| **pneumonia < 1year** | 1.59 [1.02,2.47] | .041 | 1.50 [0.98,2.30] | .061 | 1.62 [1.10,2.39] | .015 | 1.09 [0.65,1.78] | .747 | 2.45 [1.43,4.14] | <.001 | 1.38 [-0.49,3.25] | .147 |
| **CFS** | 1.14 [0.98,1.33] | .097 | 1.25 [1.09,1.44] | .002 | 1.25 [1.10,1.41] | <.001 | 1.19 [1.01,1.40] | .034 | 1.10 [0.90,1.35] | .364 | -0.00 [-0.60,0.60] | .998 |
| **CURB-65** | 1.66 [1.33,2.09] | <.001 | 1.21 [0.97,1.51] | .089 | 1.38 [1.15,1.68] | <.001 | 1.54 [1.20,1.97] | <.001 | 0.98 [0.71,1.33] | .886 | 1.17 [0.25,2.10] | .013 |
| **AP diagnosis** | 0.61 [0.34,1.08] | .099 | 0.63 [0.35,1.13] | .128 | 0.63 [0.38,1.06] | .083 | 0.52 [0.26,1.02] | .063 | 0.52 [0.23,1.13] | .106 | -2.27 [-4.69,0.15] | .066 |
| **NBM on admission** | 4.92 [2.89,8.48] | <.001 | 1.61 [0.86,3.05] | .138 | 2.84 [1.67,4.95] | <.001 | 2.52 [1.28,5.00] | .007 | 1.47 [0.63,3.29] | .357 | 0.42 [-2.00,2.84] | .732 |
| **SLT referral** | 1.12 [0.70,1.76] | .638 | 1.55 [0.98,2.45] | .062 | 1.53 [1.02,2.30] | .040 | 2.11 [1.26,3.54] | .005 | 2.22 [1.22,4.00] | .008 | 5.93 [3.99,7.87] | <.001 |

OR: odds ratio, B: regression coefficient (for continuous outcomes), 95% CI: 95% confidence interval, CALS: CRIDECO anticholinergic load scale, CFS: clinical frailty scale, AP: aspiration pneumonia, NBM: nil by mouth, SLT: speech and language therapist
